# *UBE2D1* and *COX7C* as Potential Biomarkers of Diabetes-Related Sepsis

**DOI:** 10.1155/2022/9463717

**Published:** 2022-04-11

**Authors:** Xin Wang, Lan-tao Wang, Bin Yu

**Affiliations:** Department of Emergency, The Fourth Hospital of Hebei Medical University, 12th Health Road, Shijiazhuang, Hebei 050011, China

## Abstract

Patients with diabetes are physiologically frail and more likely to suffer from infections and even life-threatening sepsis. This study aimed to identify and verify potential biomarkers of diabetes-related sepsis (DRS). Datasets GSE7014, GSE57065, and GSE95233 from the Gene Expression Omnibus were used to explore diabetes- and sepsis-related differentially expressed genes (DEGs). Gene set enrichment analysis (GSEA) and functional analyses were performed to explore potential functions and pathways associated with sepsis and diabetes. Weighted gene co-expression network analysis (WGCNA) was performed to identify diabetes- and sepsis-related modules. Functional enrichment analysis was performed to determine the characteristics and pathways of key modules. Intersecting DEGs that were also present in key modules were considered as common DEGs. Protein-protein interaction (PPI) network and key genes were analyzed to screen hub genes involved in DRS development. A mouse C57 BL/6J-DRS model and a neural network prediction model were constructed to verify the relationship between hub genes and DRS. In total, 7457 diabetes-related DEGs and 2606 sepsis-related DEGs were identified. GSEA indicated that gene datasets associated with diabetes and sepsis were mainly enriched in metabolic processes linked to inflammatory responses and reactive oxygen species, respectively. WGCNA indicated that grey60 and brown modules were diabetes- and sepsis-related key modules, respectively. Functional analysis showed that grey60 module genes were mainly enriched in cell morphogenesis, heart development, and the PI3K-Akt signaling pathway, whereas genes from the brown module were mainly enriched in organelle inner membrane, mitochondrion organization, and oxidative phosphorylation. *UBE2D1*, *IDH1*, *DLD*, *ATP5C1*, *COX6C*, and *COX7C* were identified as hub genes in the PPI network. Animal DRS and neural network prediction models indicated that the expression levels of *UBE2D1* and *COX7C* in DRS models and samples were higher than control mice. UBE2D1 and COX7C were identified as potential biomarkers of DRS. These findings may help develop treatment strategies for DRS.

## 1. Introduction

Incidence of diabetes, a major global medical concern, is increasing at an overwhelming rate. The number of individuals with diabetes, which had reached 422 million by 2014, is expected to increase to 590 million by 2035 [1]. A national representative cross-sectional study reported that the overall prevalence of diabetes in China was 12.4% and associated with being overweight or obese, increased intake of red meat, and low physical activity [2]. Although significant progress has been made in diabetes diagnostics due to advancements in molecular methods such as real-time reverse transcriptase PCR and next-generation sequencing technology [3, 4], further studies are needed to explore and improve diagnostic testing for diabetes. Diabetes mellitus (DM) is considered a major comorbidity in patients presenting with sepsis. The incidence of diabetes and diabetes-related sepsis (DRS) has been increasing parallelly. A large retrospective cohort study involving 12,321 sepsis cases in intensive care reported 3,509 (28.48%) cases of comorbid diabetes, while the in-hospital mortality associated with DRS was 14.88% [5].

Sepsis is a clinically complex, life-threatening syndrome characterized by acute organ dysfunction resulting from a dysfunctional bodily response to infection [6]. Yearly, an estimated 49 million sepsis cases and 11 million sepsis-related deaths are reported worldwide [7]. Improved sepsis diagnosis requires the development of more efficient, effective, and accessible tools and strategies that are applicable in community settings [8]. Molecular- and biomarker-based diagnostic techniques combined with traditional blood culture assays are reliable tools for managing sepsis [9, 10]. Both sepsis and diabetes share certain dysregulated immune pathways, which may induce a more destructive host response in diabetics with sepsis. Thus, immunomodulatory approaches targeting pathways shared in diabetes and sepsis may be promising therapeutic options [11]. Frydrych et al. found that individuals with type 2 diabetes (T2D) were more likely to develop fatal infections and die from sepsis owing to immune dysfunction and physiological frailty [12]. Another study indicated that altered immune response in patients with diabetes may accelerate microorganism growth and contribute to sepsis progression [13]. However, the current lack of effective biomarkers for DRS hinders the development of novel potential therapeutic targets for this common but devastating disease. Thus, exploring the relationship between diabetes and sepsis may lead to the development of novel diagnostic methods.

Gene Expression Omnibus (GEO; http://www.ncbi.nlm.nih.gov/geo) is a powerful and comprehensive international public genomics database of high-throughput resources with numerous web-based tools to support researchers in reanalyzing bioinformatics data [14]. In the present study, we identified DEGs associated with diabetes and sepsis by analyzing three mRNA expression profiles from the GEO database. Gene Ontology (GO), Kyoto Encyclopedia of Genes and Genomes (KEGG), gene set enrichment analysis (GSEA), and weighted gene co-expression network analysis (WGCNA) were used to study the molecular mechanisms underlying diabetes and sepsis. Subsequently, we constructed a PPI network of diabetes- and sepsis-related DEGs and WGCNA key modules to identify hub genes of DRS. Next, the expression levels of hub genes were validated via RT-qPCR using mouse model of DRS. Finally, a neural network prediction model was constructed to screen for potential biomarkers of DRS. These innovative explorations may assist us in developing potential novel biomarkers and effective gene-targeted treatment for diabetes, sepsis, and DRS.

## 2. Materials and Methods

### 2.1. Diabetes and Sepsis Datasets

**Figure 1:**
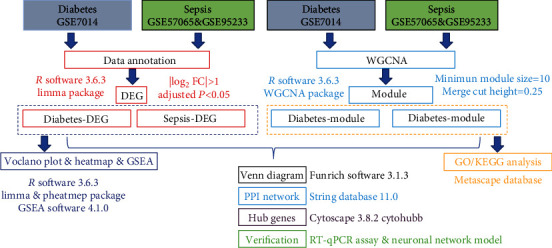
Flowchart showing data analysis approach used in this study.

We selected gene expression profiles of diabetes and sepsis from the open-access GEO database. The following keywords were used for search: “diabetes,” “sepsis,” “homo sapiens,” and “expression profiling by array.” Three investigators reviewed these datasets independently and conducted face-to-face discussions on controversial opinions. Datasets containing the gene expression profiles of blood tissue from patients with diabetes or sepsis were included. Datasets with a sample size <10, from in vitro research, or lacking a healthy control group, were excluded. Finally, we extracted a diabetes-related dataset (GSE7104) and two sepsis-related datasets (GSE57065 and GSE95233) for subsequent analysis [15–17]. All datasets were generated from the GPL 570 [HG-U133_Plus_2] Affymetrix Human Genome U133 Plus 2.0 Array. A GSE7014 dataset, including 10 DM1 biopsies, 20 DM2 biopsies, and the biopsies of six healthy individuals, was used to identify DEGs between diabetics and healthy controls. The GSE57065 dataset contained 28 patients with sepsis and 25 healthy volunteers. The GSE95233 dataset contained 51 patients with sepsis and 22 healthy volunteers. GSE57065 and GSE95233 were used to identify DEGs between sepsis patients and healthy volunteers (Table 1). The flowchart of the analysis is shown (Figure 1).

### 2.2. Data Processing and Identification of DEGs

Perl script was used to transform probe information into gene names, and background correction and normalization of datasets were performed using R package “limma” (version 3.42.0, https://bioconductor.org/packages/limma/) [18] and “affy” (version 1.72.0 https://bioconductor.org/packages/affy/) [19] provided by the open bioinformatics source platform, Bioconductor (version 3.12, http://bioconductor.org/). We adjusted the original *P*-values using the Benjamini-Hochberg method and calculated fold changes (FC) in the false discovery rate. DEGs were identified using a threshold of |log2 FC| > 1 and adjusted *P*-value <0.05 and visualized using the limma package in R software (version 3.6.3, https://www.r-project.org/). Volcano plots and heatmaps of diabetes- and sepsis-related DEGs were visualized using the “ggplot2” package (version 3.3.3, https://ggplot2.tidyverse.org/) [20] and “pheatmap” package (version 1.0.12, https://CRAN.R-project.org/package=pheatmap) in R software (version 3.6.3), respectively.

### 2.3. Weighted Gene Co-Expression Network Analysis (WGCNA)

WGCNA was carried out using the “WGCNA” package (version 1.69, https://cran.r-project.org/web/packages/WGCNA/index.html) in R software (version 3.6.3) [21]. First, a co-expression network was constructed for all the genes, and 25% genes showing the highest variance were filtered prior to further analysis. The samples were then used to create an adjacency matrix, which was then transformed into a topological overlap matrix. Genes were divided into different modules using TOM-based differences. When exploring modules, minimal gene module size was set to 30, and the threshold to merge similar modules was set to 0.25. Pearson's correlation was used to evaluate the correlation between the modules and diabetes or sepsis. The module with the highest correlation with diabetes or sepsis was identified as the module of interest for subsequent analyses.

### 2.4. Gene Set Enrichment Analysis (GSEA)

GSEA is a powerful analytical tool that interprets gene expression data and provides functional and pathway enrichment analyses [22]. In this study, GSEA was performed to explore the potential functions and molecular mechanisms underlying diabetes and sepsis. Gene annotation files, reference function sets, and all gene data of both diabetes vs. normal group and the sepsis vs. normal group were imported and analyzed using GSEA software (version 4.1.0, http://www.gsea-msigdb.org/gsea/index.jsp). Statistical significance was set at *P* < 0.05.

### 2.5. Functional Enrichment Analysis from Metascape Database

Gene Ontology (GO) analysis is a comprehensive resource of gene functions and widely used to analyze omics and related data. Kyoto Encyclopedia of Genes and Genomes (KEGG) analysis associated genomic details with higher-order functional information. Metascape (http://metascape.org) is a powerful online database that provides comprehensive analysis resources and gene list annotations [23]. In our study, GO and KEGG analyses of modules of interest were performed using the Metascape database. The threshold for pathway and process enrichment was set at a minimum overlap of 3; *P* value cutoff was 0.05, while minimum enrichment was 1.5.

### 2.6. Identification of Common Genes, Protein-Protein Interaction (PPI) Networks, and Hub Genes

FunRich (version 3.1.3, http://www.funrich.org/) is a standalone software tool that may be used to graphically depict gene analysis results [24]. First, we explored and created Venn diagrams for common genes for diabetes-DEGs, sepsis-DEGs, diabetes-WGCNA module, and sepsis-WGCNA module, using FunRich software. The STRING database (version 11.0, http://string-db.org/) is a publicly available database that provides comprehensive lists of differentially expressed genes [25]. In this study, PPI networks of common DEGs were analyzed using the STRING database (confidence score > 0.4). Subsequently, PPI networks were visualized and analyzed using Cytoscape software (version 3.8.2). To investigate the hub genes based on the PPI network, a Cytoscape plug-in CytoHubba (version 0.3) was employed to rank essential nodes in the PPI network using different topological analysis methods (closeness, radiality, betweenness, and eccentricity) [26]. The top 14 node genes were then defined and extracted from node results, ranked according to closeness, radiality, betweenness, and eccentricity. Node genes shared by these four topological analyses were identified as hub genes of interest by drawing Venn diagrams using FunRich software (version 3.1.3, http://www.funrich.org/).

### 2.7. DRS Animal Model

Twenty male C57BL/6 mice (6-8 weeks) were obtained from Beijing Huafukang Biological Technology Co., Ltd. and randomly divided into two groups: normal (*n* = 10) and DRS (*n* = 10). A mouse model of diabetes in the DRS group was induced via intraperitoneal injection of streptozotocin (60 mg/kg/d for 5 consecutive d) [27]. Sepsis was induced in mice in the diabetic group via ligation and perforation of the cecum as previously reported [28]. All mice were anesthetized with a 3% sodium pentobarbital solution. Then, a 2-cm midline laparotomy that exposed the cecum was performed using a scalpel. Then 1/3 of the junction of the ileum and cecum was ligated with 4-0 silk and punctured twice using an 18 G needle. All mice were sacrificed 24 h after the model was successfully constructed, and blood samples were collected.

### 2.8. RT-qPCR Assay

Total RNA was extracted from blood samples using TRIzol® (Beijing Biolab Technology Co., China) and reverse-transcribed using the Servicebio®RT First-Strand cDNA Synthesis kit (cat. no. G3330; Wuhan Servicebio Biotechnology Co., Ltd.) for 60 min at 42°C. The reaction was terminated by heating the mixture at 70°C for 5 min. RT-qPCR was performed using a Light Cycler® 4800 System (Roche Diagnostics) with a specific set of primers for amplifying the selected hub genes. Primers used are listed in Table 2. The thermocycling conditions were as follows: 95°C for 15 s; followed by 60°C for 60 s (total 30 cycles). The relative expression (relative quantification = 2^−ΔΔCt^, where Ct represents quantification cycle values) of each sample were calculated and is presented as a fold change of gene expression relative to the control group. GAPDH was used as an endogenous control.

### 2.9. Construction of the Neural Network Model

Both inputs and outputs of the neural network must be determined prior to performing neural network modeling. Due to the limitation of data, some input feature vectors in the disease risk determination model had to be excluded, and data pertinent to the three features of UBE2D1, COX7C, and DRS were used as input vectors to conduct limited validation of the DRS disease risk determination model; this model reflects the health status of the body to a large extent due to the relatively large impact of these characteristics.

An individual's diagnostic result corresponds to the three characteristics-based data entered, wherein regarding health status, 1 represents that the individual is positive for DRS and 0 represents that the individual is negative for DRS. To fit the data and provide the risk of DRS, the model only required a corresponding output.

### 2.10. Statistical Analyses

Data are expressed as percentages and mean ± SD. An independent-samples *t*-test was used, and where equal variances could not be assumed, the Brown-Forsythe test was performed. Pearson's rho test was performed to analyze the degrees of correlation between hub genes and DRS. The effect of the relative parameters on DRS was evaluated using multivariate linear regression analysis. All statistical analyses were conducted using SPSS software (version 24.0; IBM Corp., Armonk, NY, USA) and MATLAB (R2014a, MathWorks Inc., New Mexico, USA). Statistical significance was set at *P* < 0.05.

## 3. Results

### 3.1. Detection of DEGs

**Figure 2:**
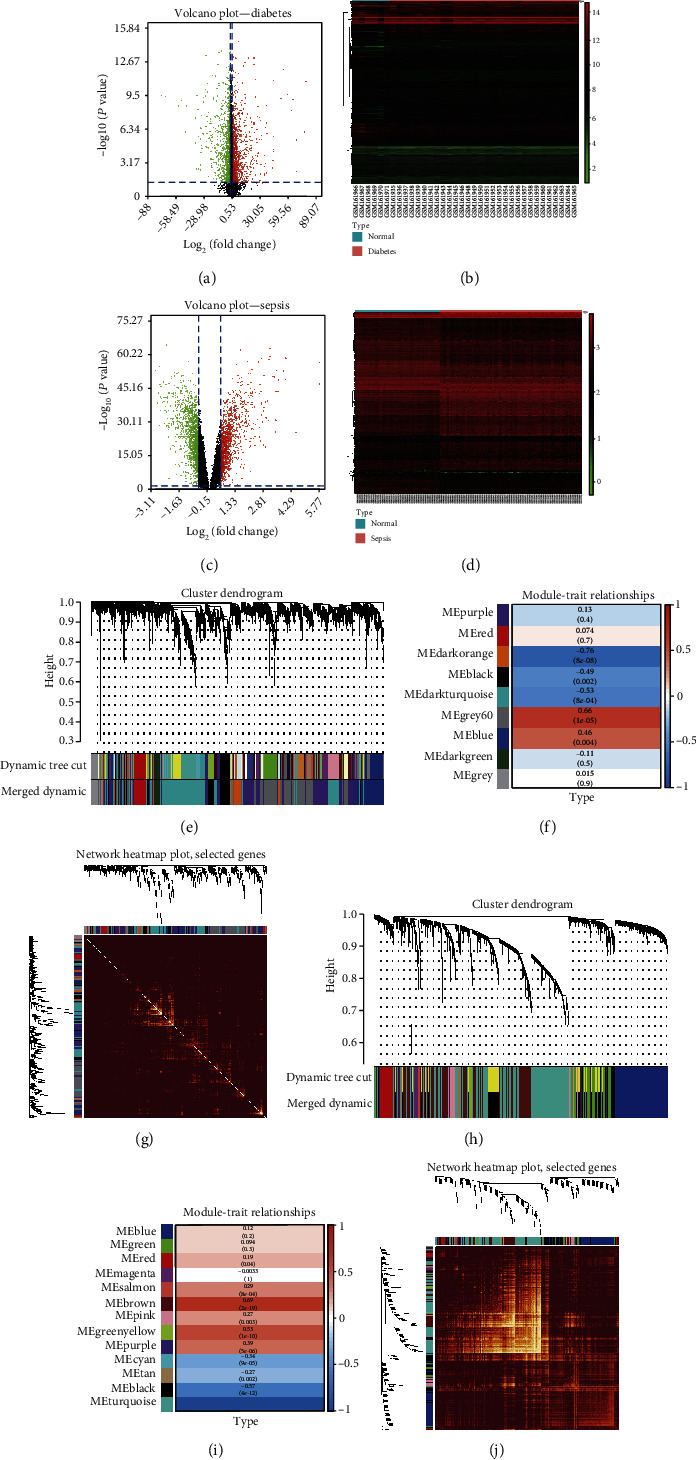
Differential expression analysis and WGCNA of genes involved in diabetes and sepsis. Volcano plots of the DEGs between diabetes vs. normal group (a) and sepsis vs. normal group (c). Heatmaps of the DEGs in diabetes series (b) and sepsis series (d). (e–g) WGCNA of the genes linked to diabetes. (e) Repeated hierarchical clustering tree of all genes. (f) The associations between clinical traits and modules. (g) Dendrogram and heatmap of genes. (h–j) WGCNA of the genes involved in sepsis. (h) Repeated hierarchical clustering tree of all genes. (i) The associations between clinical traits and modules. (j) Dendrogram and heatmap of genes.

In this study, 55764 probes and 20485 genes in the GSE7014, GSE57065, and GSE95233 datasets were determined. A total of 7,457 diabetes-DEGs were obtained, comprising 3,912 genes with upregulated expression and 3,545 genes with downregulated expression (Figures 2(a) and 2(b)). Subsequently, 2,606 sepsis-DEGs were identified, comprising 913 genes with upregulated expression and 1,693 genes with downregulated expression (Figures 2(c) and 2(d)).

### 3.2. WGCNA and Identification of Modules of Interest

For WGCNA-based analysis of the diabetes datasets, the soft thresholding power was 10. A hierarchical clustering tree of all genes in the diabetes database was constructed, and nine modules were generated (Figure 2(e)). The grey60 module was most strongly related to diabetes (Figure 2(f)). The dendrogram and heatmap of genes showed that the differences in the interactions between different modules were not significant, proving that these modules had a high degree of independence (Figure 2(g)).

The soft-thresholding power for WGCNA of sepsis datasets was 11. Furthermore, a hierarchical clustering tree of all genes in the diabetes database was produced. Thirteen modules were generated (Figure 2(h)), where the brown module correlated most with sepsis (Figure 2(i)). The dendrogram and heatmap of the genes demonstrated that these modules had a high degree of independence (Figure 2(j)).

### 3.3. Functional and Pathway Enrichment Analysis of GSEA and Metascape Database

**Figure 3:**
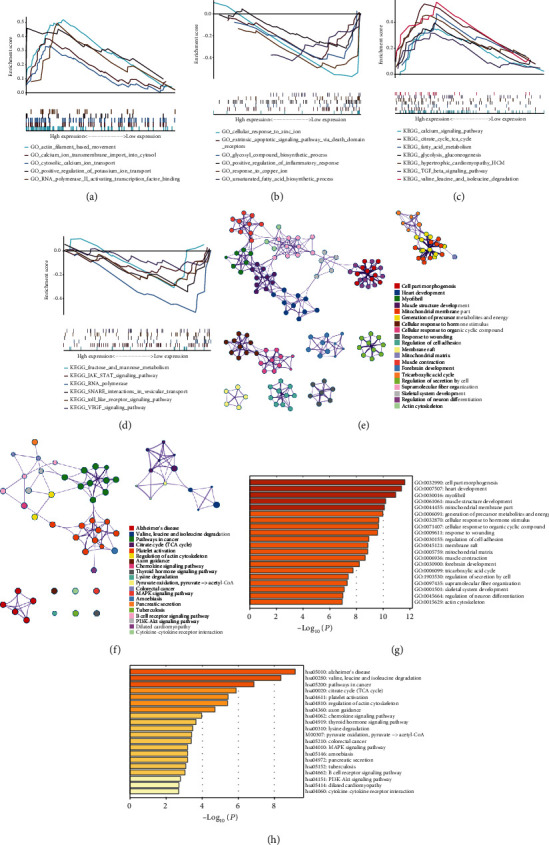
Enrichment analysis of diabetes-related datasets using GSEA and Metascape: (a–d) enrichment of GO/KEGG using GSEA; (e, f) enrichment of GO/KEGG based on cluster analyses; (g, h) enrichment heatmap of selected GO/KEGG analyses.

For diabetes-related datasets, the results of Gene Ontology analysis were significantly enriched in calcium ion transmembrane import into cytosol, cytosolic calcium transport, and positive regulation of inflammatory response, among others (Figures 3(a) and 3(b)). The results of KEGG analysis were significantly enriched in gluconeogenesis, TGF-beta signaling pathway, and JAK-STAT signaling pathway, among others (Figures 3(c) and 3(d)). The GO analysis indicated that genes from the diabetes-related grey60 module were mainly enriched in cell morphogenesis, heart development, myofibril, muscle structure development, mitochondrial membrane part, and generation of precursor metabolites and energy (Figures 3(e) and 3(g)). The results of KEGG analysis were primarily enriched for Alzheimer's disease, valine, leucine, and isoleucine degradation, cancer pathways, citrate cycle, platelet activation, and regulation of the actin cytoskeleton (Figures 3(f) and 3(h)).

**Figure 4:**
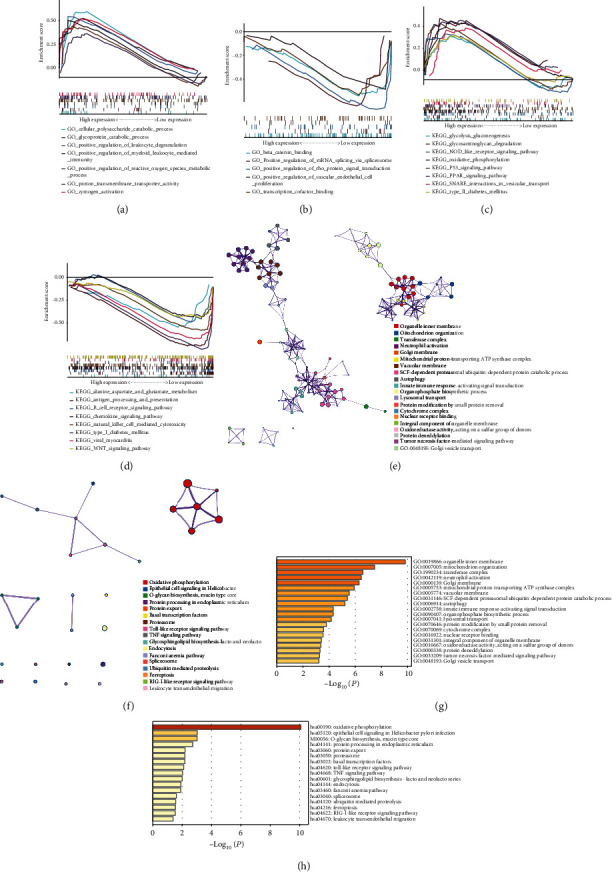
Enrichment analysis of sepsis-related datasets using GSEA and Metascape: (a–d) enrichment of GO/KEGG using GSEA; (e, f) enrichment of GO/KEGG based on cluster analyses; (g, h) enrichment heatmap of selected GO/KEGG analyses.

For sepsis-related datasets, the results of GO analysis were significantly enriched in positive regulation of leukocyte degranulation, positive regulation of reactive oxygen species, metabolic process, and regulation of vascular endothelial cell proliferation, among others (Figures 4(a) and 4(b)). The results of KEGG analysis were significantly enriched in glycosaminoglycan degradation, oxidative phosphorylation, and Wnt signaling pathway, among others (Figures 4(c) and 4(d)). The GO analysis of genes from the sepsis-related brown module showed significant enrichment in organelle inner membrane, mitochondrion organization, transferase complex, neutrophil activation, Golgi membrane, mitochondrial proton-transporting ATP synthase, etc. (Figures 4(e) and 4(g)). The enrichment results of KEGG analysis were mainly enriched in oxidative phosphorylation, epithelial cell signaling in *Helicobacter*, O-glycan biosynthesis, mucin-type core, protein processing in the endoplasmic reticulum, protein export, and basal transcription factors (Figures 4(f) and 4(h)).

### 3.4. Identification of Common Genes and PPI Networks

**Figure 5:**
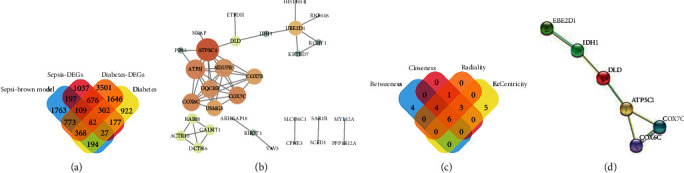
Relationship between DEGs in diabetes and sepsis series: (a) the Venn diagram of common genes between diabetes-related DEGs, sepsis-related DEGs, grey60 module and brown module; (b) PPI network of the common genes; (c) the Venn diagram of shared hub genes; (d) PPI network of shared hub genes.

We defined 82 common genes among the diabetes-DEGs, sepsis-DEGs, diabetes-related grey60 modules, and sepsis-related brown modules (Figure 5(a)). PPI networks of common DEGs were constructed using STRING and Cytoscape (Figure 5(b)), and 31 nodes and 96 edges were identified via the PPI network. Hub genes were ascertained using the following four algorithms; betweenness, closeness, radiality, and eccentricity. We then created Venn diagrams of the hub genes using the four algorithms (Figure 5(c)). *UBE2D1*, *IDH1*, *DLD*, *ATP5C1*, *COX6C*, and *COX7C* were identified as the hub genes (Figure 5(d); Table 3).

### 3.5. Results of RT-qPCR for Hub Genes

**Figure 6:**
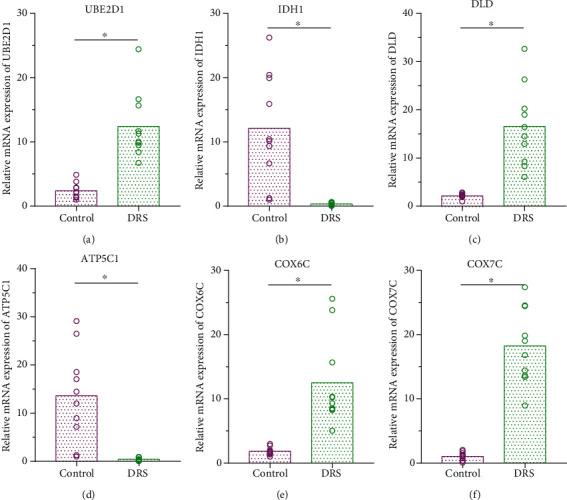
The relative expression levels of hub genes based on the RT-qPCR. The PCR results indicated that the expression levels of *UBE2D1*, *DLD*, *COX6C*, and *COX7C* in the diabetes-related sepsis group were significantly higher than those of the normal group, whereas the relative expression levels of *IDH1* and *ATP5C1* were significantly lower (*P* < 0.05).

RT-qPCR results (Figure 6) indicated that the relative expression levels of *UBE2D1*, *DLD*, *COX6C*, and *COX7C* in the DRS group were all significantly higher than those in the normal group (*P* < 0.05). However, the relative expression levels of *IDH1* and *ATP5C1* in the DRS group were significantly lower than those in the normal group (*P* < 0.05).

### 3.6. Correlation Analysis of the Hub Genes and DRS

**Figure 7:**
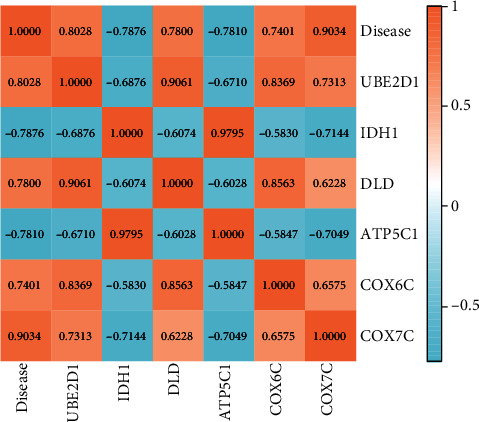
Heatmap showing strong correlations between the hub genes and diabetes-related sepsis (DRS). DRS was associated with the relative expression of *COX7C* (*P* < 0.001; *R* = 0.9034). In addition, a strong relationship is seen between *UBE2D1* expression and DRS (*P* < 0.001; *R* = 0.8028).

Pearson's rho analysis showed a strong relationship between hub genes' expression and DRS (*P* < 0.05). DRS was associated with the relative expression levels of *COX7C* (*P* < 0.001, *R* = 0.9034), *COX6C* (*P* < 0.001, *R* = 0.7401), *ATP5C1* (*P* < 0.001, *R* = −0.7810), *DLD* (*P* < 0.001, *R* = 0.78), and *IDH1* (*P* < 0.001, *R* = −0.7876). In addition, a strong relationship was observed between *UBE2D1* expression and DRS (*P* < 0.001, *R* = 0.8028). Furthermore, the heatmap showed strong correlations between the hub genes and DRS (Figure 7).

### 3.7. The Effect of Correlative Genes on DRS Based on Multiple Linear Regression Analysis

To confirm that the significant risk factors had an impact on DRS, we analyzed DRS and associated risk factors. A multivariate linear regression model indicated that when all other variables were held at any fixed value, DRS remained associated with *COX7C* expression (*β* = 0.652, *P* < 0.001). In addition, no collinearity issues were detected among other factors (Table 4).

### 3.8. *UBE2D1* and *COX7C* Were Strongly Correlated with DRS according to the BP Neural Network

**Figure 8:**
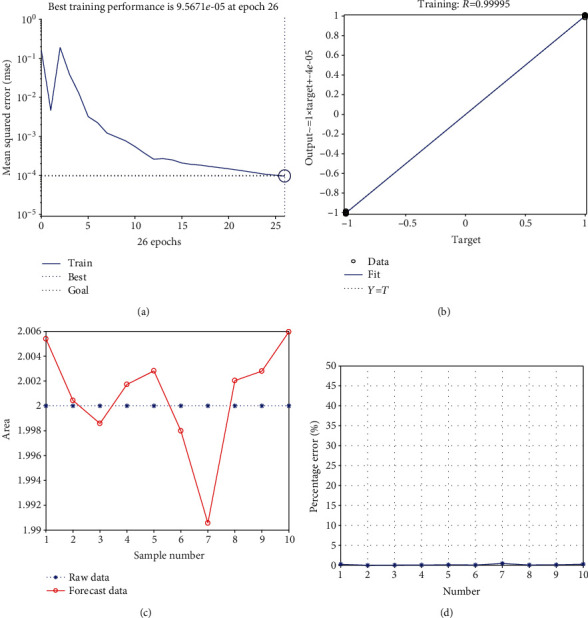
Correlation between candidate genes and diabetes-related sepsis based on the neural network: (a) the best training performance; (b) the relativity of training; (c) the comparison chart of training results; (d) the error analysis diagram.

The best training performance was 9.5671e-05 at epoch 26 (Figure 8(a)), with a relativity of 0.99995 (Figure 8(b)). The model verified this result, and there were no significant differences between the predicted and actual values (Figures 8(c) and 8(d)). Based on these results, we speculate that the levels of *UBE2D1* and *COX7C* expression may act as predictive indices for DRS.

## 4. Discussion

Incidence of diabetes and sepsis has reached epidemic proportions worldwide. Mounting evidence has established diabetes as a risk factor for sepsis. However, the molecular mechanisms underlying such association have not been fully elucidated. Bioinformatics offers an ideal means for screening large gene expression datasets, which may lead to a better understanding of the potential links between these two diseases [29–31]. Zou et al. performed a comprehensive bioinformatics-based gene analysis to identify potential biomarkers and therapeutic targets pertaining to atrial fibrillation-related stroke, and their results showed that atrial fibrillation and stroke were related and that four hub genes were significantly associated with novel biomarkers of atrial fibrillation-related stroke [29]. Santiago et al. compared the blood transcriptomes of patients with mild cognitive impairment, Alzheimer's disease (AD), and advanced AD with those of individuals afflicted with T2D and revealed shared and unique pathways and potential therapeutic targets, which suggested that T2D may play a role at different stages of AD by disrupting various molecular pathways during preclinical as well as more advanced stages of the disease [30]. Rahman et al. applied a high-throughput network-based quantitative pipeline using agnostic approaches to identify abnormally expressed genes in both T2D and neurological diseases, as well as some of the shared molecular pathways that underpin T2D and neurological disease interactions [31]. Similar to these studies, our investigation of co-expressed genes, aimed at gaining a better understanding of the relationship between diabetes and sepsis, revealed *UBE2D1* and *COX7C* as potential biomarkers and therapeutic targets for DRS.

In the present study, we performed a comprehensive bioinformatic analysis, which combined WGCNA and DEG analyses to detect hub genes in crucial modules of the co-expression network, which allowed the mining of more valuable gene data. The diabetes and sepsis datasets were downloaded from the GEO database and reanalyzed to identify modules that correlated most with DRS. GO and KEGG analysis of the diabetes-grey60 module and the sepsis-brown module were carried out to identify the potential molecular mechanisms associated with the genes in the module. Interestingly, the chemokine signaling pathway and the B cell receptor signaling pathway were enriched in KEGG analysis of the diabetes-grey60 module. Thus, we hypothesized that genes in the grey60 module may play a crucial role in the immune system. Hyperglycemia, inflammation, and subsequent impairment of the immune system in diabetics may increase their susceptibility to severe infections and sepsis [32, 33]. Daryabor et al. reported that dysfunctional immune responses induced by diabetes may reduce the ability to control invading pathogens, resulting in patients with diabetes being more susceptible to infections and complications [33]. Moreover, KEGG analysis indicated that the sepsis-brown module was enriched in the TNF signaling pathway and leukocyte transendothelial migration, which substantiated the crucial role played by the immune system in sepsis. Therefore, immunotherapy may be a promising early treatment strategy against DRS.

In this study, six hub genes (*UBE2D1*, *IDH1*, *DLD*, *ATP5C1*, *COX6C*, and *COX7C*) were identified based on PPI network analysis. UBE2D1 (ubiquitin-conjugating enzyme E2D1) is a member of the E2 ubiquitin-conjugating enzyme family, which plays an essential role in the degradation of dysfunctional or aged proteins. Ubiquitin-conjugating enzyme splice variants are reportedly associated with increased maternal fasting plasma glucose [34]. Azzam et al. identified *UBE2D1* as a genetic risk factor for diabetic retinopathy in Emirati patients with T2DM [35]. In addition, proteins possessing E2 ubiquitin enzyme activity are reportedly associated with sepsis-related mortality [36]. Therefore, ubiquitin-conjugating enzymes may form key nodes between diabetes and sepsis. IDH1 (isocitrate dehydrogenase 1) catalyzes oxidative decarboxylation of isocitrate to 2-oxoglutarate. IDH1 expression is upregulated during the differentiation of brown adipocytes, which are a novel therapeutic target for T2D [37]. Thus, IDH1 may play a vital role in the occurrence and development of DRS. DLD (dihydrolipoamide dehydrogenase), a member of the class I pyridine nucleotide-disulfide oxidoreductase family, acts as a dehydrogenase that plays a key role in pyruvate oxidation and tetrahydrofolate metabolism and serves as a potential marker of diabetes in human myocytes [38]. Su et al. also reported that DLD may be a useful metabolic biomarker of sepsis [39]. Thus, we propose DLD as a potential and valuable biomarker of DRS. *ATP5C1* (ATP synthase, H+ transporting, mitochondrial F1 complex, gamma polypeptide 1) is a mitochondria-related gene. Mitochondria-related genes, such as *ATP5C1* and *TIMM9*, promote mitochondrial biogenesis, which is beneficial for the treatment of diabetes [40]. COX6C (cytochrome c oxidase subunit 6C) and COX7C (cytochrome c oxidase subunit 7C) are subunits of cytochrome c oxidase that transfers electrons from cytochrome c to oxygen. Interestingly, expression of COX6C is significantly upregulated in rat diabetic nephropathy models [41]. Moreover, Zhang et al. demonstrated that telmisartan ameliorates damaged kidney function in diabetic rats by regulating mitochondrial oxidative phosphorylation [42]. Therefore, we hypothesized that mitochondrial oxidative phosphorylation may play an essential role in DRS. These results suggest that the hub genes identified in our study may be pivotal regulators of the pathophysiological processes of DRS.

In the current study, functional and pathway enrichment analyses were performed to explore the potential functions and signaling pathways of genes shared among the diabetes-DEGs, sepsis-DEGs, diabetes-related grey60 module, and sepsis-related brown module. Notably, the calcium signaling pathway was significantly enriched in the GSEA. Many cellular functions are reportedly regulated by the calcium signaling pathway. Wang et al. indicated that altering Ca2+-dependent signaling pathways helped regulate the activities of oxidative metabolism and mitochondrial function [43]. Sabatini et al. demonstrated that the upregulation of calcium signaling pathways in insulin-producing *β*-cells may initiate many signaling pathways and stimulate vesicle exocytosis [44]. Long-term activation of these pathways, which is not conducive to *β*-cell health, plays an important role in T2D. Moreover, Zhang et al. indicated that nNOS (neuronal NOS) may inhibit TNF-alpha (myocardial tumour necrosis factor-alpha) in cardiomyocytes in response to LPS (lipopolysaccharide) treatment [45]. Peng et al. reported that the Ca2+ signaling pathway is activated during sepsis, indicating that blocking the calcium signaling pathway may protect the heart, liver, and kidneys from sepsis-induced damage [46]. Thus, we hypothesized that Ca2+-dependent signaling pathways may play a key role in diabetes and sepsis. However, these findings need to be verified in the future studies.

An animal modal and a neuronal network prediction model were constructed to validate the relationship observed between these hub genes and DRS. A RT-qPCR assay revealed that the expression of *UBE2D1*, *DLD*, *COX6C*, and *COX7C* was significantly upregulated compared with controls, which was consistent with the result of our bioinformatics analysis. In addition, the results of the neural network prediction model indicated that the levels of UBE2D1 and COX7C expression could be predictive indices of DRS.

Similar to most research-based bioinformatic analyses, our study was also beset by some limitations. Firstly, common diabetes- and sepsis-related DEGs may not be completely equivalent to potential biomarkers of DRS, and thus, the biomarkers identified in this study may require validation using DRS datasets. Thus, we plan to collect blood samples from animal models of DRS or DRS patients and conduct sequencing experiments to verify our results. Secondly, although we verified the association between the identified hub genes and DRS via RT-qPCR, the reliability of our conclusions may be significantly improved by using larger sample sizes for experimentation as well as clinical verification. Thirdly, mounting evidence indicates that biological sex differences play a role in the risk and clinical presentation of diabetes and sepsis [7, 47]. Our study did not conduct sex or sex subgroup analyses due to a lack of essential clinical information.

## 5. Conclusions

In the present study, we performed bioinformatics analyses and verification experiments and identified *UBE2D1* and *COX7C* as potential biomarkers of DRS. Our findings provide new insights and directions for the development of treatment strategies for DRS. Nevertheless, these findings need further validation via additional experimental and clinical research.

## Figures and Tables

**Table 1:** Summary of diabetes and sepsis microarray datasets from GEO database.

	Series	Platform	Affymetrix GeneChip	Healthy controls	Patients with diabetes/sepsis
1	GSE7014	GPL570	[HG-U133_Plus_2] Affymetrix Human Genome U133 Plus 2.0 Array	6	30
2	GSE57065	GPL570	[HG-U133_Plus_2] Affymetrix Human Genome U133 Plus 2.0 Array	25	28
3	GSE95233	GPL570	[HG-U133_Plus_2] Affymetrix Human Genome U133 Plus 2.0 Array	22	51

**Table 2:** Primers sequences used for RT-qPCR.

Primer	Sequence (5′–3′)
GAPDH-hF	TGAAGGTCGGAGTGAACGGAT
GAPDH-hR	CGTTCTCAGCCTTGACCGTG
UBE2D1-hF	GAATAAATGTTAGCTGTCCCTA
UBE2D1-hR	AGGATGAGGCTGGAAATG
IDH1-hF	AGGCTCTGCTGATTCTTT
IDH1-hR	TTCTTAACTTTGCGATGC
DLD-hF	CCTTGGGTAAATCAGAAA
DLD-hR	AGGCCAACATCATTGTAT
ATP5C1-hF	TGAGCAGAGTGCCAGGAT
ATP5C1-hR	CGGGTACGGTTGAATGTC
COX6C-hF	TTGCTCTGGCTAGGACTT
COX6C-hR	CAGATTTGACATCGCATTA
COX7C-hF	CAGGAGTTCCAGACCAGCCT
COX7C-hR	TGGCCAGGCTGGTCTGGAAC

**Table 3:** Summary of hub genes.

Symbol	Description	Function
ATP5F1C	ATP synthase F1 subunit gamma	GO:0042776 mitochondrial ATP synthesis coupled proton transport; GO:0015986 ATP synthesis coupled proton transport; GO:0042407 cristae formation
DLD	Dihydrolipoamide dehydrogenase	GO:0061732 mitochondrial acetyl-CoA biosynthetic process from pyruvate; GO:0006086 acetyl-CoA biosynthetic process from pyruvate; GO:0006554 lysine catabolic process
IDH1	Isocitrate dehydrogenase (NADP(+)) 1	GO:0006740 NADPH regeneration; GO:0060696 regulation of phospholipid catabolic process; GO:0006097 glyoxylate cycle
UBE2D1	Ubiquitin conjugating enzyme E2 D1	GO:0035666 TRIF-dependent toll-like receptor signaling pathway; GO:0002756 MyD88-independent toll-like receptor signaling pathway; GO:1902916 positive regulation of protein polyubiquitination
COX7C	Cytochrome c oxidase subunit 7C	GO:0006123 mitochondrial electron transport, cytochrome c to oxygen; GO:0042775 mitochondrial ATP synthesis coupled electron transport; GO:0042773 ATP synthesis coupled electron transport
COX6C	Cytochrome c oxidase subunit 6C	GO:0006123 mitochondrial electron transport, cytochrome c to oxygen; GO:0042775 mitochondrial ATP synthesis coupled electron transport; GO:0042773 ATP synthesis coupled electron transport

**Table 4:** The effects of correlated genes on diabetes-related sepsis based on multiple linear regression analysis.

Factors	Diabetes-related sepsis		
	*β* ^b^	*P*-value	VIF
*UBE2D1*	-0.163	0.501	8.420
*IDH1*	-0.103	0.810	26.748
*DLD*	0.462	0.058	7.495
*ATP5C1*	-0.054	0.899	25.847
*COX6C*	-0.031	0.861	4.456
*COX7C*	0.652	<0.001	2.770

*β*: parameter estimate.

## Data Availability

The datasets used and/or analyzed during the current study are available from the corresponding author on reasonable request.
